# Decomposing compounds enables reconstruction of interaction fingerprints for structure-based drug screening

**DOI:** 10.1186/s13321-022-00592-w

**Published:** 2022-03-15

**Authors:** Melissa F. Adasme, Sarah Naomi Bolz, Ali Al-Fatlawi, Michael Schroeder

**Affiliations:** Biotechnology Center (BIOTEC), CMCB, Technische Universitat Dresden, Tatzberg 47-49, 01307 Dresden, Germany

**Keywords:** Fragments, Binding mode, Structural data, Non-covalent interactions, Interactions fingerprint

## Abstract

**Background:**

Structure-based drug repositioning has emerged as a promising alternative to conventional drug development. Regardless of the many success stories reported over the past years and the novel breakthroughs on the AI-based system AlphaFold for structure prediction, the availability of structural data for protein–drug complexes remains very limited. Whereas the chemical libraries contain millions of drug compounds, the vast majority of them do not have structures to crystallized targets,and it is, therefore, impossible to characterize their binding to targets from a structural view. However, the concept of building blocks offers a novel perspective on the structural problem. A drug compound is considered a complex of small chemical blocks or fragments, which confer the relevant properties to the drug and have a high proportion of functional groups involved in protein binding. Based on this, we propose a novel approach to expand the scope of structure-based repositioning approaches by transferring the structural knowledge from a fragment to a compound level.

**Results:**

We fragmented over 100,000 compounds in the Protein Data Bank (PDB) and characterized the structural binding mode of 153,000 fragments to their crystallized targets. Using the fragment’s data, we were able to artificially reconstruct the binding mode of over 7,800 complexes between ChEMBL compounds and their known targets, for which no structural data is available. We proved that the conserved binding tendency of fragments, when binding to the same targets, highly influences the drug’s binding specificity and carries the key information to reconstruct full drugs binding mode. Furthermore, our approach was able to reconstruct multiple compound-target pairs at optimal thresholds and high similarity to the actual binding mode.

**Conclusions:**

Such reconstructions are of great value and benefit structure-based drug repositioning since they automatically enlarge the technique’s scope and allow exploring the so far ‘unexplored compounds’ from a structural perspective. In general, the transfer of structural information is a promising technique that could be applied to any chemical library, to any compound that has no crystal structure available in PDB, and even to transfer any other feature that may be relevant for the drug discovery process and that due to data limitations is not yet fully available. In that sense, the results of this work document the full potential of structure-based screening even beyond PDB.

**Graphical Abstract:**

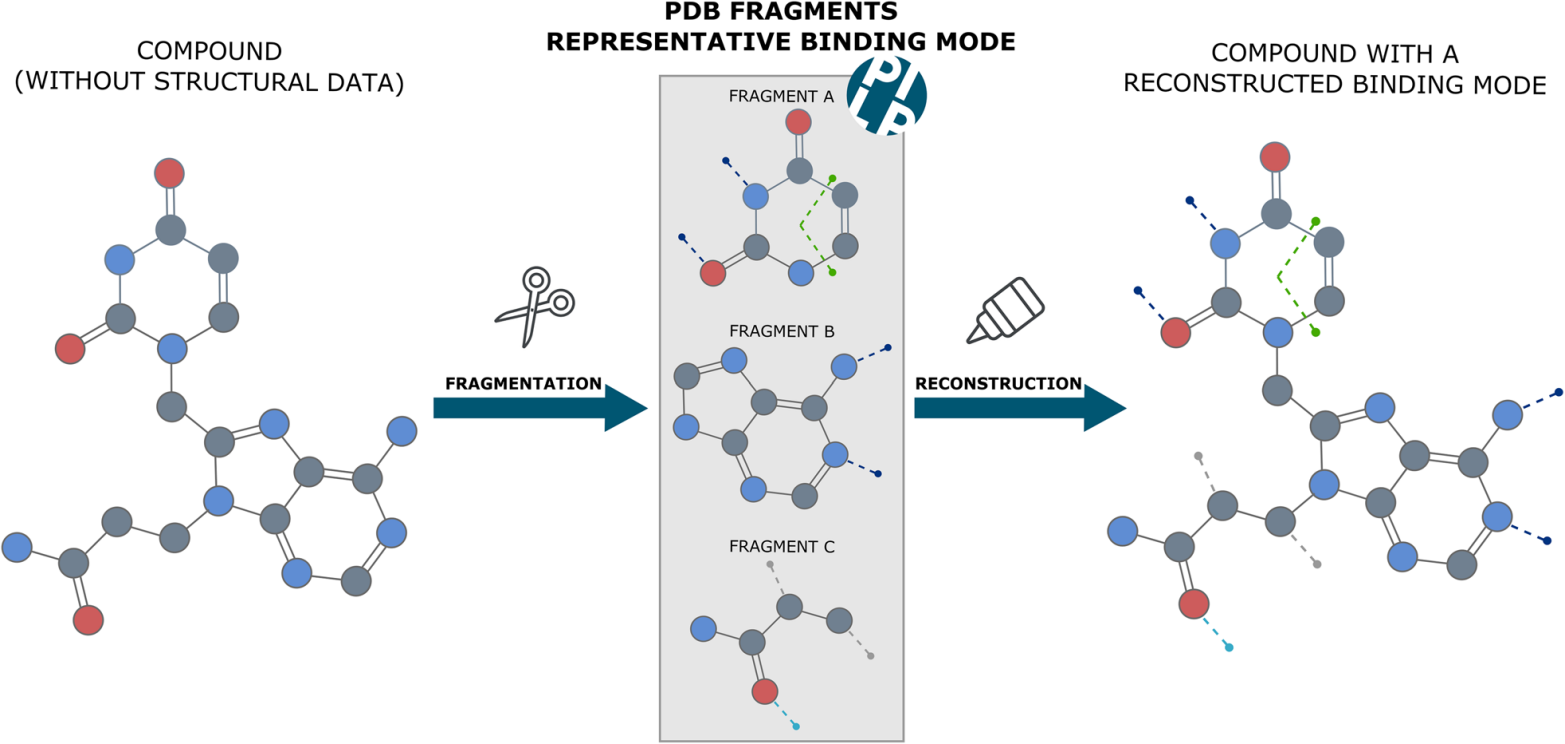

**Supplementary Information:**

The online version contains supplementary material available at 10.1186/s13321-022-00592-w.

## Background

Drug repositioning seeks the identification of new purposes for already existing drugs. The benefits of this approach are a decreased risk of failure, less time required for the development of a drug, and reduced costs [1]. This makes drug repositioning an attractive alternative to conventional drug discovery and development.

Structure-based drug repositioning exploits the 3D structure of proteins to characterize the binding mode of drugs to their protein targets under an energetic/geometrical perspective. This knowledge leads to the screening and discovery of novel drug-targets links serving as repurposed opportunities. Several techniques work under the structural concept, e.g. docking, binding site prediction, pharmacophore-based screening, interaction similarity screening, among others [2]. The in-silico screening based on 3D interaction data studies the binding mode similarities of drugs and identify novel targets for the repositioning candidates. Many studies have previously exploited the concept of interactions fingerprints on drug repositioning [3–7]. However, a more recently fingerprinting technique based on the Protein-Ligand Interactions Profiler (PLIP) tool [8] has been successfully applied for the repositioning of Amodiaquine as a cancer treatment [9], for the identification of ibrutinib as a new inhibitor of the autoimmune-related target VEGFR2 [10], for the identification of repurposed drugs as Chagas treatments [11], and the prediction of novel LRRK2 inhibitors [12], among others.

The starting point of any structure-based drug repositioning pipeline is the collection of structural data describing the geometrical conformations of drug compounds binding to crystallized targets. Currently, with more than 170,000 structures, PDB is estimated to cover the vast majority of the known drug targets (about 92%) [13], with more than 52,000 different protein sequences, and most of the structures (more than 60%) in complex with biologically relevant ligands [14]. However, notwithstanding the great amount of structural data available and despite many years of continuous effort, not all therapeutically relevant protein families are equally represented in structural databases. In fact, according to Khafizov et al., 60% of known protein families in the Pfam database still lacked structural characterization [15]. For instance, with over 20,000 entries, enzymes are the structurally most populated family by far, while only a handful has been resolved for GPCRs. Moreover, out of the millions of drug compounds contained in chemical libraries such as Pubchem or ChEMBL, the vast majority of them do not have structures to crystallized targets and are therefore impossible to characterize from a structural perspective. Taken all together, the availability of structural data remains a clear limitation to structure-based drug repositioning.

Alternatively, the concept of building blocks offers a novel perspective on the problem. A drug compound is a complex of small chemical blocks, called fragments, which confer the relevant properties to the drug [16]. Fragments have, in principle, a high proportion of functional groups involved in protein binding, and many of them precisely fit the target sub pockets. Moreover, due to their reduced size and complexity, fragments allow an efficient exploration of protein binding sites [17]. In a previous study, Kozakov et al. showed that fragments coinciding with low-energy hot spots tend to have conserved binding modes [18]. Later on, Drwal et al. performed a large scale analysis of the PDB, aiming for a deep understanding of fragment binding to ligandable targets [19]. It was observed that the binding modes of fragments and their drug-like superstructures binding to the same protein are mostly conserved. In a more recent study, Giordanetto et al. [20] carried out a comprehensive analysis on the deposited protein structures with bound fragment hits, suggesting that attractive interactions, such as Hydrogen bonds, water bridges, and coordination bonds to catalytic metal ions constitute a recurring stabilizing feature of the majority of the fragment-hit complexes. All previous studies suggest that small chemical changes in the fragment are tolerated without alteration of the fragment's binding mode. In that sense, it seems relevant to explore the structural data at a fragment level.

Given the highlighted relevance of molecular fragments in the drug discovery process, it seems interesting to explore their molecular properties and binding mode conservation from a structural perspective. However, most of the aforementioned studies have been conducted in a relatively limited space. For instance, Drwal et al. work was constrained to fragments crystallized as small molecule ligands in PDB structure, meaning the narrowed set is biased towards crystallographers’ research interests. In this work, we characterize the binding of molecular fragments in all the PDB drugs, we define a structural metric to evaluate the binding mode conservation of fragments, and we later use such data to reconstruct the binding mode of full drugs without structural data. We seek to evaluate how feasible it is to transfer the structural knowledge from a fragment level to a drug level and thereby expand the scope of structure-based drug repositioning and other approaches that rely on structural characterization of drugs.

**Fig. 1:**
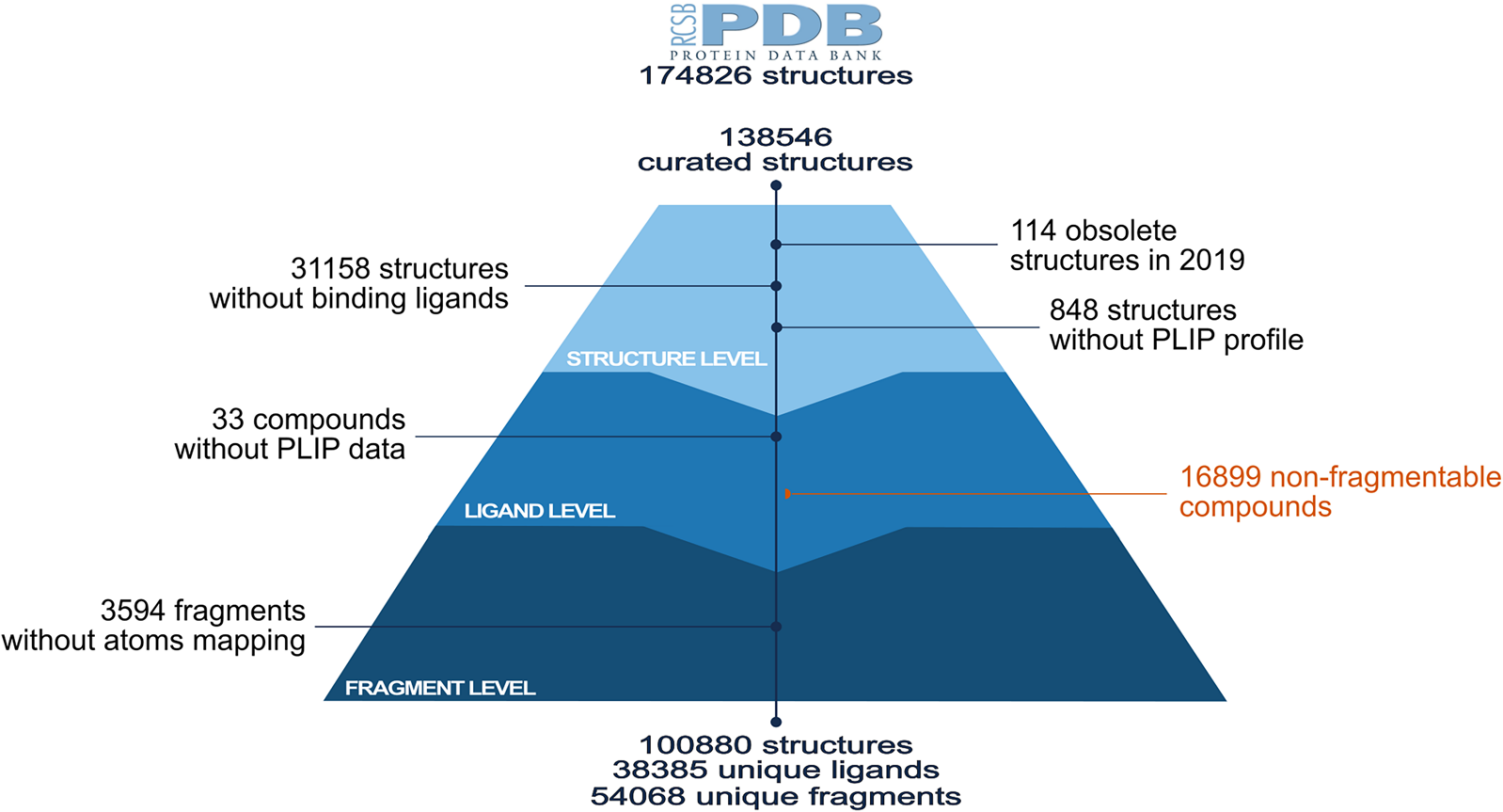
PDB fragments data set construction. From the top starting point of the curated structures in PDB until the final resulting fragments, passing over the the three layers representing the extraction data levels: structures, ligands, and fragments. The lost of data is accounted and explained for each layer in the pyramid

**Fig. 2:**
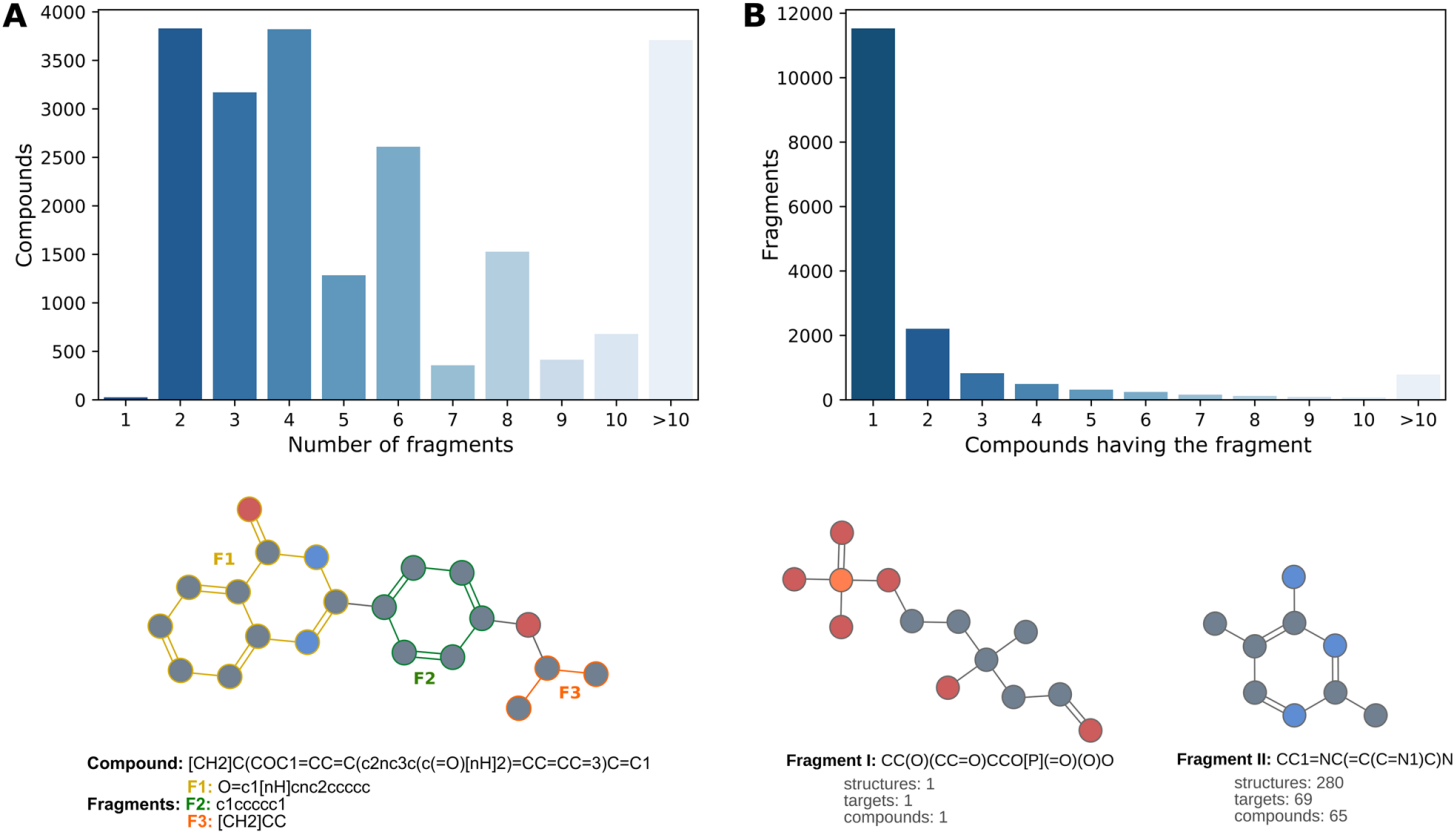
Fragmentation of PDB compounds. **A** The bar plot shows the amount of PDB compounds by the number of fragments obtained after fragmentation. Most of the compounds are fragmented from 2 to 6 fragments. The compound at the bottom is an example of fragmentation with three fragments F1, F2 and F3. **B** The bar plot shows the frequency of fragment among the compounds (x axis shows on how many compounds is a fragment found). Most of them are present in just one compound, but others are highly frequent. The fragment I at the bottom left is present in only one compound, whereas the fragment II on the right is part of 65 different compounds

**Fig. 3:**
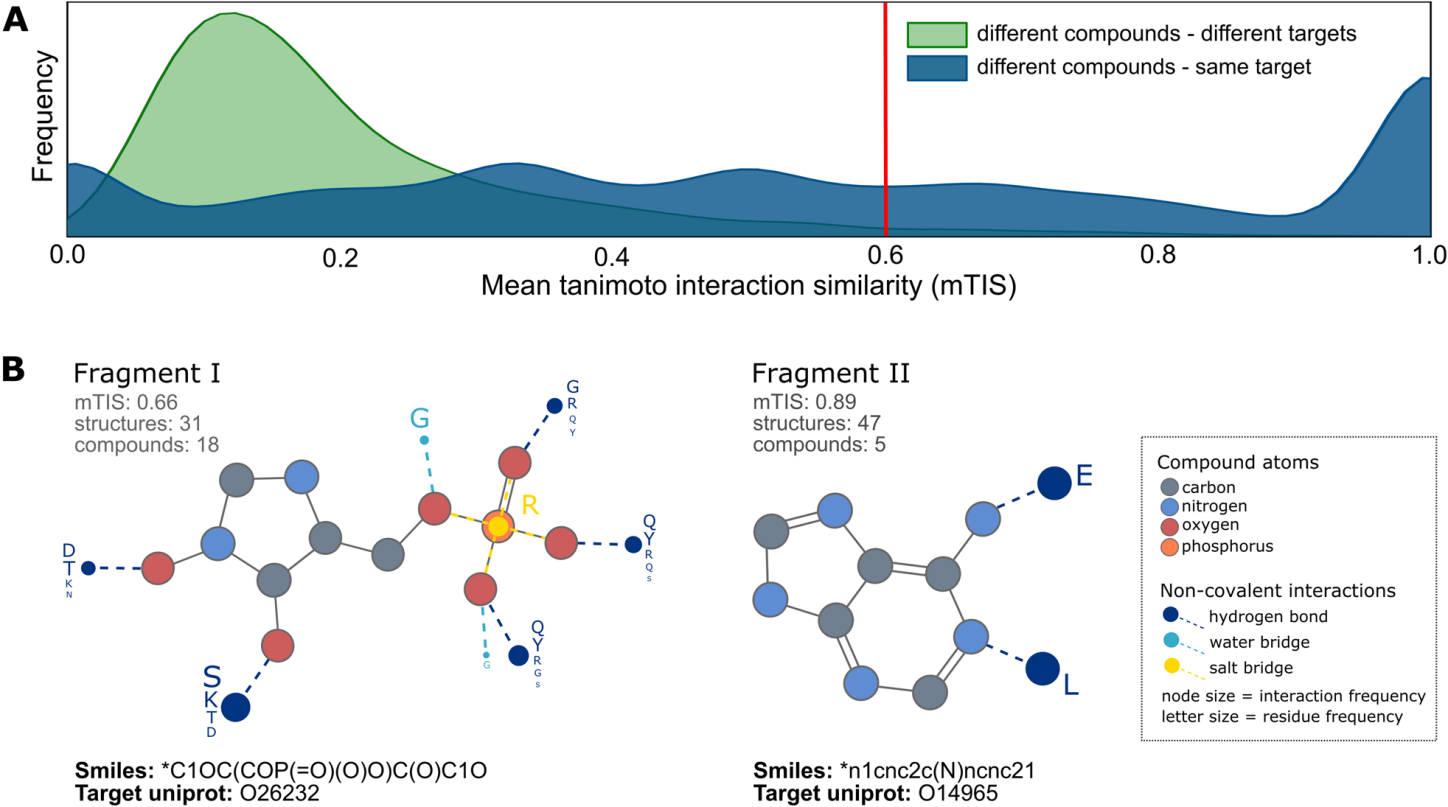
Binding mode conservation of PDB fragments. The binding mode of the fragments has been characterized with PLIP interactions and represented as binary fingerprints. **A** For each fragment a mean Tanimoto Interaction Similarity (mTIS) was calculated and plotted as frequency under two scenarios: fragments in different compounds binding different targets (green) and fragments in different compounds binding to the same target (blue). **B** The fragment I is part of 18 different compounds binding to the same target (O26232) and it has a relatively conserved binding mode with a mTIS of 0.66. The interactions displayed (dashed colored lines) are highly conserved among the different structures, with variations only in the target residues (letters). The fragment II is part of 5 different compounds binding to the same target (O14965) and it has a highly conserved binding mode (mTIS of 0.89) with two hydrogen bonds to the target residues Glutamate (E) and Leucine (L)

## Results and discussion

### Fragments from the PDB ligands fragmentation

In order to expand the previous analysis carried out by Drwal et al. [19], fragments and their structural features were directly extracted from all molecules in PDB. Using the RECAP algorithm with tree leaves, 100,880 PDB structures and 38,385 ligands were successfully fragmented into 54,068 molecular fragments (Fig. 1).

When manually analyzing more in-depth some of the fragments obtained, it was observed that many of them are large fragments mostly derived from steroids, porphyrins, and staurosporin analogues, among others. Many others are amino acids, nucleotide analogues, or a few sugars. For the latter, specifically, amino sugars, which are usually very soluble and have several groups capable of interacting with the target protein in a usually well defined 3D structure. At a less frequent level, were observed organic fragments with hetero atoms capable of interacting with the target proteins, specifically with kinases. Overall, the number of fragments per ligand obtained after fragmentation varies from ligand to ligand (Fig. 2A). Most of the compounds were fragmented from 2 to 10 fragments. Only 26 compounds are formed by one unique fragment, whereas more than 6000 compounds lead to more than ten fragments. This latter reflects the complex chemical design of some compounds. In general, it is expected that the number of fragments in a compound is directly influenced by the size of the same. Meaning that larger compounds are expected to have more fragments and vice versa. However, a Pearson correlation of $-0.01$ shows no correlation between both features.

At the same time, Fig. 2B shows that PDB fragments tend to be unique among the PDB compounds and are present in one compound only, which is explained by the complex diversity of structures in the chemical space and the reduced chemical space in PDB. Nonetheless still, more than 10,000 fragments are present in at least two different compounds. Such distribution directly correlates with that of World Drug Index (50 K molecules) in the original RECAP publication [21]. Examples of both cases are illustrated in Fig. 2B where the fragment on the left bottom is part of just one compound binding to one unique target, whereas the fragment on the right bottom is a substructure of 65 different compounds binding to 69 different targets. The unique essence of the left fragment could be attributed to its chemical structure. Despite the phosphate group being a frequent functional group among the chemical compounds, the rest of the fragment’s chemical structure is relatively rare. On the other hand, the recurrent appearance of the fragment at the right is due to its properties as a pyrimidine derivative compound. It is well known that the pyrimidine ring system has wide occurrence in nature [22], and therefore, the same applies to all its derivatives.

### Binding mode conservation of PDB fragments

In a similar manner, as performed in the analysis by Drwal et al., the binding mode conservation of PDB fragments was further explored. It is well known that the PDB data is unbalanced and usually biased towards biologically relevant proteins or over-represented compound scaffolds resulting from lead optimization. To make a fair estimation of binding mode conservation, the PDB fragments data must be adequately filtered and homogenized, considering certain criteria (see Methods). As mentioned before, one unique fragment can be a substructure of multiple different compounds, in multiple different PDB structures (or complexes). In order to estimate how conserved the binding mode for a given fragment is, all the PDB complexes containing such fragments were compared in terms of interactions fingerprints.

The non-covalent interactions of all PDB compounds were calculated using the PLIP tool [8] with standard settings. Following, the non-covalent interactions for the PDB fragments were encoded into a binary fingerprint, which was constructed considering only the interactions mediated by the fragment atoms. The interactions were encoded in a simple fingerprint of 500 bins, totally ignoring the geometrical features of the interactions and instead focusing on the types of interaction in the involved functional groups and in the interacting residues (see Methods section for more details). It might be expected that the size of the fragments relates to the number of observed non-covalent interactions patterns, thus influencing its binding mode. However, a Pearson correlation of 0.6 shows a moderate positive correlation between the size of the fragments and the number of interactions patterns displayed in the binding mode. The binding mode similarity of PDB fragments is measured by calculating a pairwise Tanimoto Interaction Similarities (TIS) of fragments' interaction fingerprints (see Additional file 1). Figure 3A shows the mTIS (mean Tanimoto Interaction Similarity) score of each PDB fragment evaluated under two criteria in the context of targets and compounds: the same fragment in all different compounds superstructures binding all kinds of targets (green curve) and the same fragment as part of different compounds binding always to the same target pocket (blue curve).

The red line is the proposed threshold at mTIS=0.6 to define binding mode conservation, which is based on literature [19]. Thus, fragments on the right of the red line are considered to have a conserved binding mode. Whereas fragments on the left, the opposite. Overall, most PDB fragments (96%) display a non-conserved binding mode when compared without considering the ligand superstructure and the target they bind to (green curve). Nonetheless, when the fragments' binding mode is compared among different compounds binding to the same target (curve blue), the majority (56%) shows a more conserved tendency.

Furthermore, since the fingerprints are dependent on the binding site residues, it was observed that minor variations on the target’s interacting residues among the different structures might affect the final mTIS scores, as observed in the examples in Fig. 3B. Fragment I is part of 18 different compounds binding to the same target (O26232). Although the interactions displayed (dashed coloured lines) are highly conserved among the different structures, they have small variations in the target residues (letters) of the different structures, leading to a relatively conserved binding mode with an mTIS of 0.66. On the other hand, Fragment II is part of 5 different compounds binding to the same target (O14965), and it has a highly conserved binding mode (mTIS of 0.89) with two hydrogen bonds to the target residues Glutamate (E) and Leucine (L). In general, there is a conserved nature of the fragments' binding mode, which suggests they can be used to extend the limits of structure-based drug repositioning by offering a different perspective to explore the binding mode of full drugs.

### Binding mode reconstruction approach

The concept of molecular fragments and their conserved binding modes have been further exploited to overcome the limitations on data availability. Overall, the reconstruction of drugs’ binding mode seeks to transfer the structural knowledge from molecular fragments to full molecules.

The performance of the approach was evaluated in a PDB subset of compound-target pairs (see Additional file 2). Given that the compounds in the PDB subset have available structures and, thus, an actual PLIP fingerprint describing their binding mode, it is possible to check how similar the reconstructed fingerprints are compared to the real ones. The PDB subset includes all compound-target complexes found up to the second level (Ligand level) of the pyramid in Fig. 1, along with the non-fragmentable compounds that were excluded at the fragmentation stage.

The reconstruction pipeline was applied to the PDB subset, trying different combinations of the modifiable thresholds, i.e. the compound’s proportion and the fragments’ binding mode conservation. To avoid bias in the validation, the binding mode (fingerprint) of a compound-target complex is reconstructed without using the structural data from the complex itself. Meaning the construction of representative binding mode of fragments does not take into consideration the fingerprint of the compound-target complex to be reconstructed. Furthermore, the quality of reconstruction was evaluated for each threshold combination. To this purpose, a Reconstruction Similarity Score (RSS) was defined as the mean of all the reconstructed-original fingerprint pairs similarities, which was calculated using the simple Tanimoto Similarity approach (see Methods section for more details).

**Table 1:** Reconstruction of PDB compound-target binding mode at different thresholds

BM conserv.	Compound proportion					
	0.5	0.6	0.7	0.8	0.9	1.0
0.5	6325^.45^	2806^.59^	1513^.65^	1175^.67^	1023^.69^	1023^.69^
0.6	4493^.49^	1581^.66^	762^.72^	589^.73^	527^.77^	527^.77^
0.7	2625^.52^	866^.71^	310^.77^	210^.77^	148^.84^	148^.84^
0.8	1662^.57^	494^.77^	162^.86^	102^.86^	66^.90^	66^.90^
0.9	1174^.63^	368^.76^	128^.89^	58^.90^	22^.95^	22^.95^
1.0	1134^.70^	368^.85^	128^.88^	58^.89^	22^.95^	22^.95^

The numbers refer to the amount of compound-target binding mode reconstructed at each threshold combination. The superscript indicate the quality of the reconstruction in terms of mean RSS, where the closest to 1.0 the better the reconstruction is. Overall, there is trade off between amount of data and quality of the reconstruction

**Table 2:** Reconstruction of ChEMBL compound-target pairs at different thresholds

BM conserv.	Compound proportion					
	0.5	0.6	0.7	0.8	0.9	1.0
0.5	7686	1737	202	48	34	34
0.6	5344	991	83	11	10	10
0.7	3844	651	49	7	6	6
0.8	2531	353	24	2	1	1
0.9	2100	279	20	1	0	0
1.0	2028	271	19	1	0	0

The numbers refer to the amount of compound-target pairs successfully reconstructed at each threshold combination

Table 1 summarizes the validation results, with the number of compound-target pairs that were reconstructed for each given threshold combination. The superscript represents the quality of the reconstruction given by the mean RSS previously calculated. The table reflects a clear trade-off between the amount of data and the quality of the reconstruction. The more restrictive the thresholds are, the less data is obtained from the reconstruction pipeline. For instance, considering the extreme case at which all the fragments of a compound have structural data (full proportion of 1.0) and only fragments with a full conserved binding mode (BM conservation of 1.0) are used, only 22 PDB compound-target pairs were reconstructed.

**Fig. 4:**
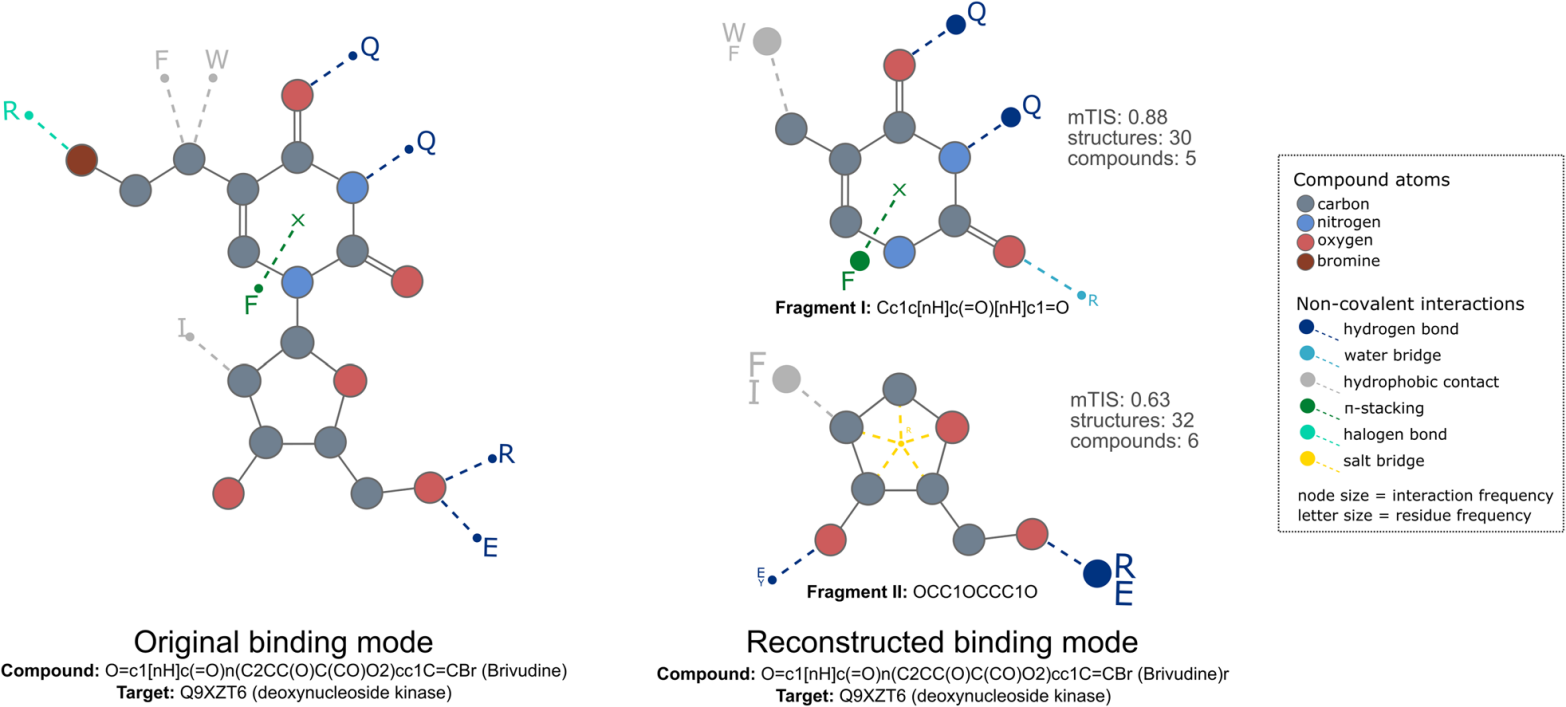
An example of binding mode reconstruction. The binding mode of the compound brivudine binding to the target deoxynucleoside kinase was reconstructed with an RSS = 0.66. On the left, the original binding mode of the complex (2VQS:BVD:C:1210) is represented and on the right the reconstructed binding mode based on the fragments. Both fragments have a relatively high binding mode conservation with an mTIS of 0.88 and 0.63. The interactions look almost alike in both cases, except for the water bridge in Fragment I and the salt bridge and the hydrogen bond in Fragment II

**Fig. 5:**
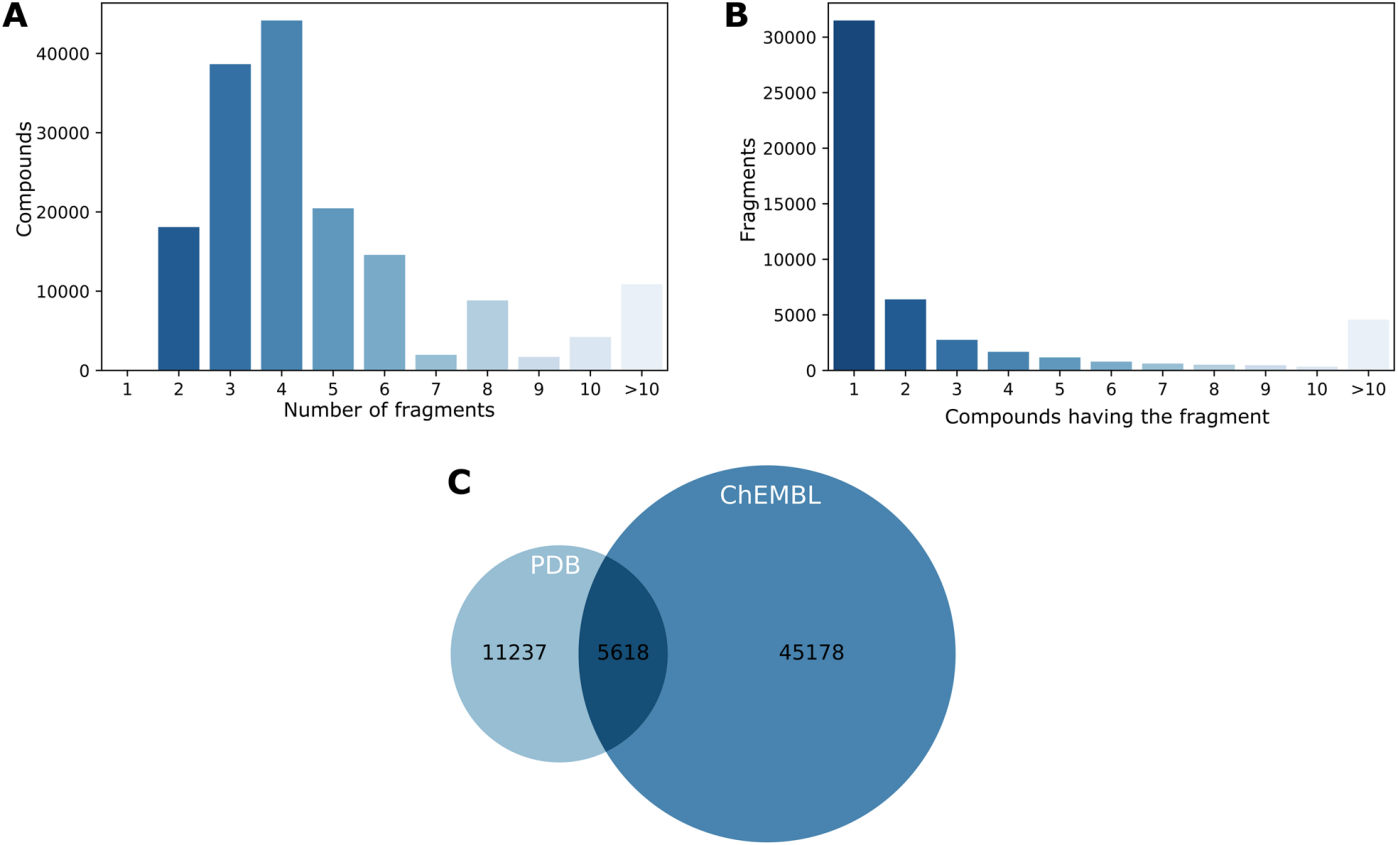
Fragmentation of ChEMBL compounds. **A** The bar plot shows the amount of ChEBML compounds by the number of fragments obtained after fragmentation. Most of the compounds are fragmented from 2 to 6 fragments. **B** The bar plot shows the frequency of fragments among the compounds. Most of them are unique for one compound, but others are highly frequent. **C** The venn diagram shows the overlapping fragments between both sources PDB and ChEMBL

**Fig. 6:**
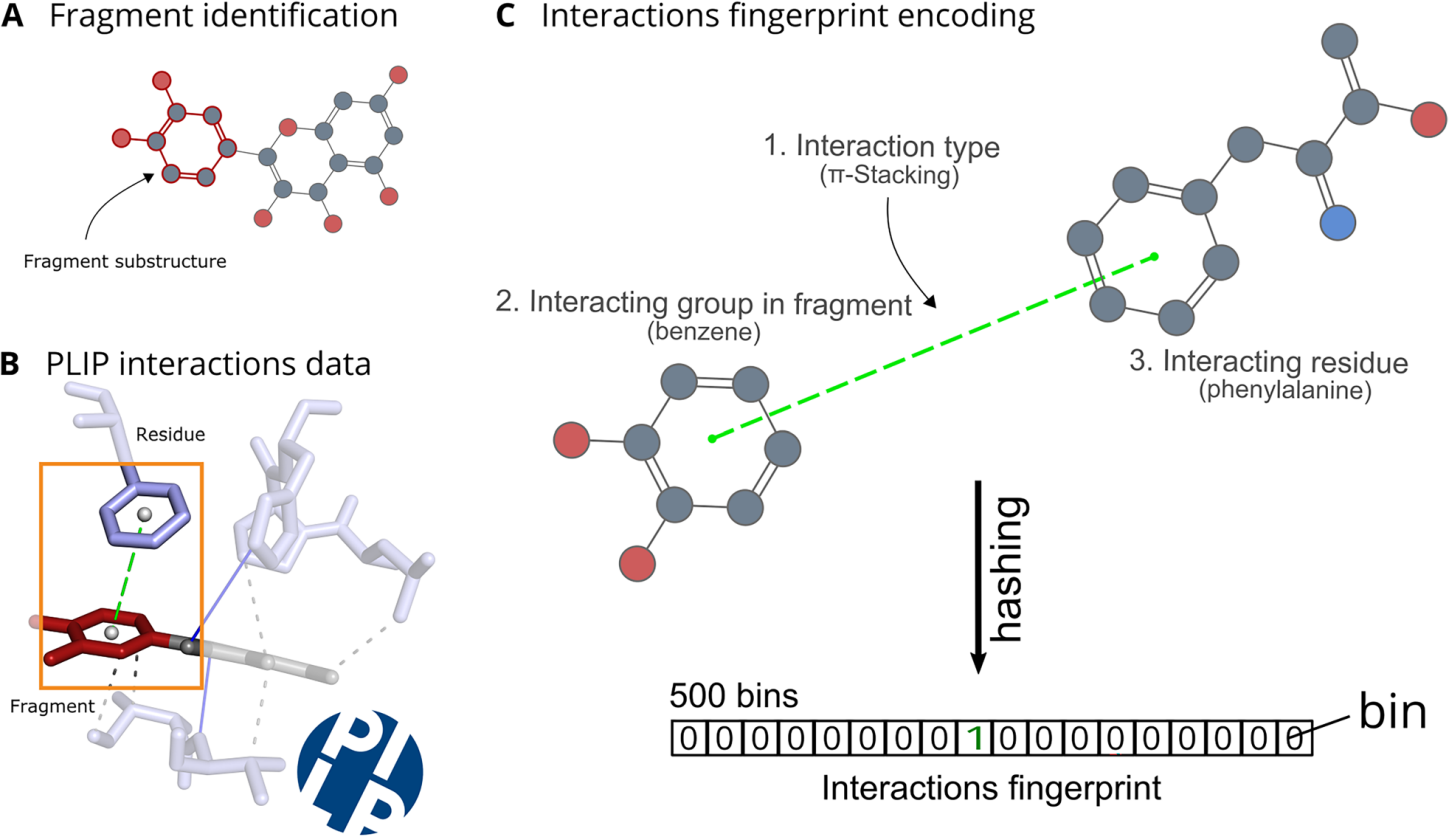
Fragments binding mode encoding. Fragments binding mode encoding. **A** Fragments are obtained from the PDB compounds. **B** The fragments atom are processed with the PLIP tool for the detection of non-covalent interactions and the generation of an interactions profile. **C** For each interaction detected in the binding mode of a fragment, three features were combined: (1)the interaction type, (2) the interacting functional group in the fragment, and (3) the interacting residue of the protein target. Each of these features combination is hashed (between 1 and 500) and encoded in a fingerprint of 500 bits

**Fig. 7:**
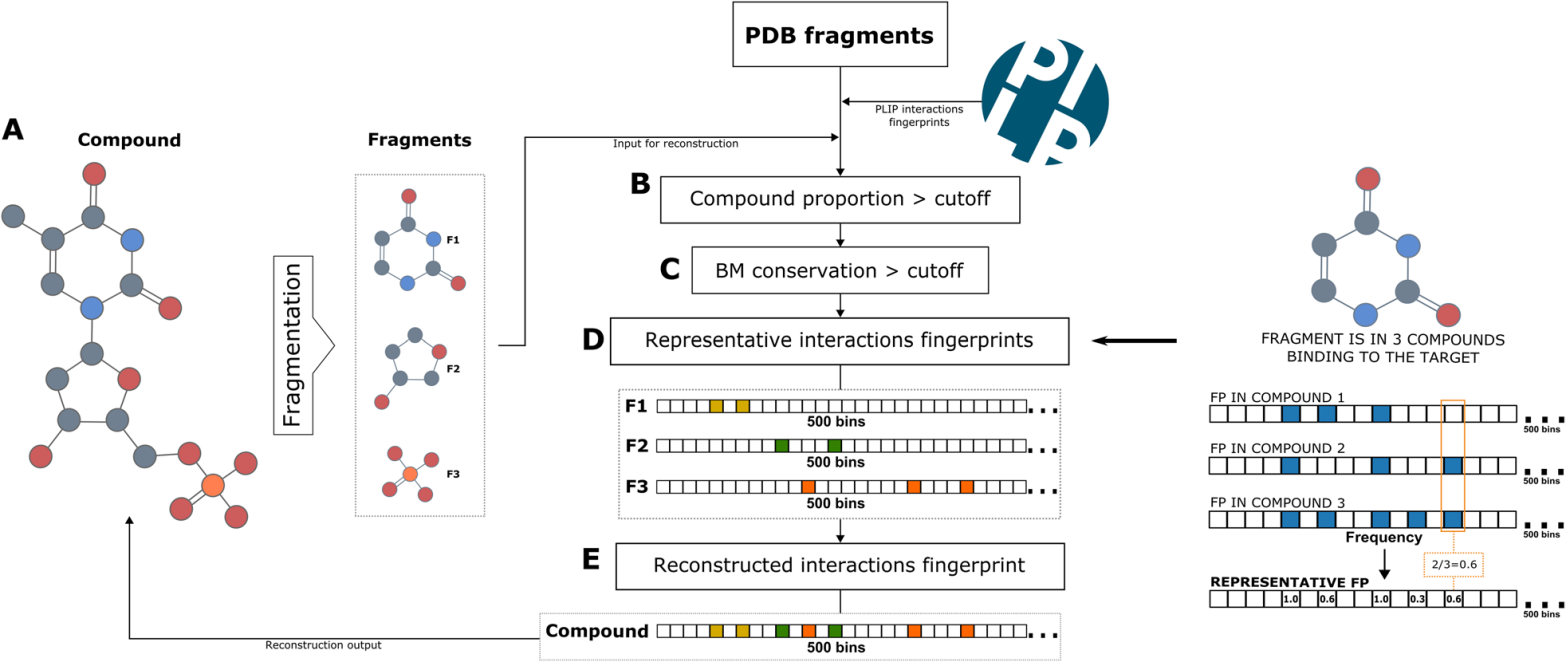
Compounds binding mode reconstruction with PDB fragments. The PDB fragments binding mode are characterized with PLIP and represented as binary interactions fingerprints. A compound without structural data is fragmented and its fragments binding mode is extracted from the PDB fragments dataset. The fragments must cover the minimum compound proportion, they must meet the size (mw) thresholds and they must meet the minimum binding mode conservation. Only then, a representative interactions fingerprint is calculated for each independent fragment to later be all merged into one interactions fingerprint representing the binding mode of the full compound

Figure 4 shows an example of the validation set at optimal thresholds: Compound proportion = 0.6 and BM conservation = 0.6, with an RSS = 0.66 when comparing the original binding mode and the reconstructed one. In the example, the drug brivudine binds to one of its targets, the deoxynucleoside kinase, with a specific set of non-covalent interactions (left). Such interactions patterns have been thoughtfully studied in a previous study [9], therefore it is a good example to evaluate the reconstruction pipeline. Brivudine can be usually fragmented into three molecular fragments: Cc1c[nH]c(=O)[nH]c1=O, OCC1OCCC1O, and Br. However, since Br has not enough structural data to characterize its binding mode conservation (less than five compounds), it is not part of the selected fragments subset and thus, not considered in the reconstruction. Nonetheless, the remaining fragments have a relatively high binding mode conservation with an mTIS of 0.88 and 0.63 (BM conservation > 0.6) which ultimately leads to a compound proportion of 2/3 = 0.66 (Compound proportion > 0.6). The binding mode of Fragment I to the deoxynucleoside kinase target was constructed using structural data of 5 different compounds in 30 different structures. The fragment exhibits a highly conserved set of non-covalent interactions, i.e. two hydrogen bonds, one hydrophobic contact, and one $\pi$-stack, which are frequent among the different structures and are always in contact with the same target residues. However, there is also a less frequent water bridge displayed in a few structures making contact with arginine (R), and it is the reason for the mTIS = 0.88. On the other hand, Fragment II was constructed using structural data of 6 different compounds in 32 different structures. It has two highly frequent interactions among the structures: a hydrogen bond always in contact with arginine or glutamic acid and a hydrophobic contact with phenylalanine or isoleucine. However, it also has another hydrogen bond, and a salt bridge displayed only in a few structures, which confers it its mTIS = 0.66, defining a more variable binding mode than fragment I. Even though none of the fragments has a perfectly conserved binding mode (mTIS = 1.0), the reconstruction turned out to be successful as it fairly represents the relevant interactions defining the binding mode of brivudine to deoxynucleoside kinase.

Overall, the validation results evidence the complexity behind the process. Although, in general, fragments tend to have a highly conserved binding mode, most of them never reach the perfect conservation of 1.0 mTIS, which comes hand to hand with the binding mode variability under special binding environments. On the other hand, the compound proportion limitation reflects the still unmet necessity of a representative structural space covering a diverse set of molecular fragments. Nevertheless, the approach was able to reconstruct multiple compound-target pairs at optimal thresholds and with high similarity (RSS) to the real fingerprints.

### Binding mode reconstruction on ChEMBL data set

ChEMBL is a manually curated database of bioactive molecules with drug-like properties [23]. The aim of ChEMBL is to collect chemical, bioactivity and genomics data to aid the translation of genomic information into effective new drugs. To date, the database contains about 2.1 million compounds, 14K biological targets, and more than 17.2 million compound-target activity assays. The large chemical space available in ChEMBL, makes it the best option to explore compounds so far not crystallized (not found in PDB) to which the proposed approach could be applied.

For this purpose, the ChEMBL dataset was explored and filtered according to several criteria (see Methods), which yielded a ChEMBL subset with a total of 264,033 compound-targets pairs. The compounds in such pairs were then fragmented with the RECAP algorithm, displaying a similar tendency to the PDB compounds regarding to the number of fragments found in each compound and the frequency of the fragments among the different compounds (see Fig. 5A, B for more details). Subsequently, the reconstruction pipeline was applied to the ChEMBL subset trying different combinations of the modifiable thresholds (see Additional file 3).

Table 2 summarizes the results, with the number of compound-target pairs that were successfully reconstructed with each given thresholds combination. The numbers in the table show a similar trade-off between the amount of data and the quality of the reconstruction. The stricter the thresholds are, the less data is obtained from the reconstruction pipeline. For instance, considering the extreme case at which all the fragments of a compound have structural data (full proportion of 1.0), and only fragments with a full conserved binding mode (BM conservation of 1.0) are used, no compound-target pair in ChEMBL was reconstructed. However, as the thresholds are relaxed, more and more reconstructions are possible.

From the proteins point of view, the majority of them (23%) belongs to the category of kinases (EC 2.7), followed by a 14% of peptidases (EC 3.4), a 6% of proteins acting on Ester Bonds (EC 3.1), a 5% of glycosyltransferases, and a 23% of proteins belonging to any other 31 groups, whereas the remaining set could not be classified to any EC number. In general, the reconstruction using PDB fragments is able to cover a small proportion of ChEMBL compounds, which demonstrates the little overlap between the PDB chemical space and giant chemical libraries such as ChEMBL (see Fig. 5C). As previously mentioned, some fragments are over-represented in PDB, whereas others barely appeared in one PDB compound. RECAP and the many other tools developed under the same basis have extensively tested the fragmentation process in multiple chemical libraries such as ChEMBL. The fact that certain fragments are found just in one PDB compound, does not directly mean that such fragments are not common among the large chemical libraries, but rather implies that there is not enough data in PDB to cover the real chemical space. The above clearly limits the reconstruction process, as the approach only uses fragments with a conserved binding mode. Therefore, no binding conservation score can be calculated if a fragment appears in less than 5 different PDB compounds. Thus, the fragment can not be used in the reconstruction. As an ultimate solution, the reconstruction constrictions could be slightly relaxed in order to avoid the aforesaid issues.However, the results should be analysed under a more permissive perspective and several uncertainties should be taken into consideration. In another aspect, it is surprising that more than 10,000 fragments are indeed part of PDB compounds but are not found in ChEMBL. Although it should be kept in mind that the ChEMBL data set was filtered prior to the fragmentation process to contain only compound-targets pairs that are not in PDB, it is expected to observe such fragments in other compounds as part of different complexes. On the contrary, the numbers suggest that they are rather unique or have not been well explored.

Computational structural approaches can provide definite insights into molecular recognition and predict binding with high confidence. The most direct technique is the chemical similarity approach, which exploits the chemical properties of the ligands, assuming that compounds with similar scaffolds will fit within the same target pockets [24]. In a similar manner, binding site similarity approaches, exploit the assumption that some protein cavities might present a similar pharmacological profile and hence, accommodate the same ligands[25]. However, they have to deal with the noise produced by the flexible chains present in the protein cavity. The methods described above tend to be focused either on the protein pocket or on the molecular properties of the ligands. Consequently, as a dependant of such properties, there is a tendency to stay within a limited scope of structurally or functionally related proteins and drugs with high similarity to the existing treatments. Integration of protein–ligand interaction profiles has recently come into research and may offer a solution to this problem. They can grasp the essence of binding sites, ignore amino acids not involved in binding, and take a more uncoupled viewpoint from the chemical structures of proteins and ligands.

On the other hand, Docking has proved to be an useful technique able to predict the orientation of a ligand into a cavity of a target protein including estimation of the binding affinity [26]. Although the technique is very well- defined and widely used, its predictive nature makes itprone to a high false-positive rate, and there are still some clear limitations. On the contrary, the reconstruction of drugs binding mode is a knowledge-based approach able to extract the binding mode properties of known structures and transfer such information to a fragment level, to be later used and reconstruct full ligands.

Taken all together, the transfer of structural information is a promising technique that could be applied to any chemical library or even more specific to any compound that has no crystal structure available in PDB. As a proof of concept, in this work, we focused our efforts on transferring the non-covalent binding modes of fragments to full drugs by using binary fingerprint representations. However, in principle, the concept of building blocks allows the transfer of any other feature, e.g. covalent bonds.

## Methods

### Fragmentation of PDB molecules

For this purpose, 138,546 curated PDB structures (to date 07.10.2020) were processed and analyzed with OpenBabel v3.0.0 [27] for the detection of small molecule compounds and their atom coordinates. The RECAP [21] algorithm was used to fragment the compounds’ SMILES (Simplified Molecular-Input Line-Entry System) and to explore their fragment space. The algorithm is implemented and distributed by the open-source RDKit v2019.09.1 with the fragmentation option for the tree leaves only. The latter ignores the option to construct fragments by merging smaller ones, leading to a reduced set of fragments avoiding redundancy of data.

For more details, Fig. 1 illustrates the resulting PDB fragments data set, where each layer of the pyramid depicts the loss of data due to different reasons. The greatest loss at the structure level is due to more than 30,000 structures without a binding ligand. Similarly, about 17,000 compounds have none of the RECAP cleavage rules; therefore, no fragments could be obtained from them. Most of such compounds cannot be fragmented because they are already fragments that were crystallized as ligands (as studied in Drwal et al. analysis [19]). Nonetheless, to keep the uniformity of the data, they were excluded from this analysis. Finally, the major loss of data at the fragment layer is due to impossible atom mapping between the original PDB file and the generated fragment molecule, given the chemical inconsistencies caused by the fragmentation process. Without such mapping, it is impossible to trace back the structural information from the PDB, and therefore, the aforesaid cases cannot be further analyzed.

### Binding mode conservation

#### Binding mode characterization

The non-covalent interactions of all PDB compounds were calculated using the Protein Ligand Interaction Profiler (PLIP) [28] with standard settings. The PDB structures without a PLIP profile and compounds without PLIP data (no interactions detected) were removed from the data set. Additionally, the PLIP non-covalent interactions for the PDB fragments were encoded into 500 bins fingerprint, which was constructed considering only the interactions mediated by the fragment atoms and encoding the types of interaction in the involved functional groups and in the interacting residues as demonstrated in Fig. 6.

#### Filtering the fragments data set

To avoid bias from over represented proteins and compounds in PDB, the RECAP algorithm considers fragments as small as one atom, e.g. the oxygen molecule. However, according to the definition of fragments, they are usually within the range of 40 < MW < 300 (Additional file 4: Fig. S1). Moreover, as shown in Fig. 2B, many fragments are a substructure of just one unique compound or only a few different targets. In such cases, the binding mode conservation can not be properly estimated. Overall, the PDB fragments data set has a mean of 7.8 in respect to the different compounds of which a fragment is part of, and 12.1 for the number of different protein targets they bind to (Additional file 5: Fig. S2). Considering the above mentioned, a fragments subset has been defined with fragments at the given molecular weight range, being a substructure of at least five different compounds, and binding to at least ten different proteins. In addition, to deal with the over-representation of some fragments compared to others, a maximum of 500 PDB complexes per fragment have been selected. If there are more than 500 complexes available in the data set for a given fragment, then its complexes are grouped by unique pairs of protein UniProt ID and compound InChIkey and only one is randomly selected as the representative complex of the pair.

#### Binding mode similarity calculation

In order to estimate how conserved the binding mode for a given fragment is, all the PDB complexes containing such fragments must be compared in terms of interactions fingerprints. The binding mode similarity of fragments is measured by calculating a pairwise Tanimoto Interaction Similarities (*TIS*) of fragments interaction fingerprints. In other words, for two protein–ligand complexes (*C*1 and *C*2) having the same fragment as substructure, the *TIS* is calculated as follow:

1 $$\begin{aligned} TIS_{C1,C2}=\frac{C1bins \cap C2bins}{C1bins+C2bins-C1bins\cap C2bins}. \end{aligned}$$

*C*1*bins* refers to the number of activated bins in the interactions fingerprint of *C*1, *C*2*bins* the activated bins in *C*2, and *C*1*bins* $\cap$ *C*2*bins* is the number of bins activated in both *C*1 and *C*2. Following, the mean of all TIS (mTIS), obtained from the pairwise similarities of a given fragment, was calculated as the score for evaluating the binding mode conservation. Finally, the mTIS of PDB fragments was evaluated under three criteria in the context of targets and compounds: the same fragment in all different compounds superstructures binding all kinds of targets, the same fragment in the same compound superstructure binding to different targets, and the same fragment as part of different compounds binding always to the same target pocket. Targets were differentiated by UniProt ID, whereas compounds by InChIkey.

### Binding mode reconstruction

Considering that the approach’s main goal is to reconstruct the binding mode of different drugs using the fragments’ structural data, a target-based reconstruction pipeline has been developed based on the category “different compounds - same target”. In other words, the binding mode of a compound can be reconstructed only for a given target, and the reconstruction considers only the structural data of fragments binding to that specific target. In order to achieve a high quality reconstruction of the binding modes, only fragments with a conserved binding mode were selected for this purpose. Since the binding mode conservation of PDB fragments was evaluated under different conditions than the state of the art study, the subset of fragments was selected with a slightly more permissive threshold at mTIS = 0.5 than the one proposed by Drwal et. al. [19], leading to a total number of 26,840 fragments-targets pairs. The fragments subset is used to reconstruct the compounds according to the following target-based pipeline:

1. *Compound fragmentation:* compounds are fragmented with the RECAP leaves algorithm using the compounds’ SMILES and default settings (Figure 7A). The fragments resulting from the fragmentation are further scanned within the PDB fragments subset. Given the target-based nature of the approach, only the structural data of fragments binding to the specific target will be further considered. If at least one of the fragments has available data in the PDB fragments subset, the approach continues. Otherwise, it is impossible to reconstruct the compound.
2. *Compound’s proportion:* Given that the PDB chemical space covers only a limited part of the ChEMBL chemical space, it is expected that many fragments have no structural data nor binding mode defined. Therefore, an optional threshold (Compound proportion) has been introduced at this step of the reconstruction (Fig. 7B), to define the minimum number of fragments considered enough to emulate the binding mode of the full compound. For instance, for the compound in Fig. 7A, a compound proportion > 0.5 would require that at least 2 of the 3 fragments are in the PDB fragments subset. If the proportion is lower than the cutoff, then the reconstruction is not possible.
3. *Binding mode conservation:* The fragments subset has been constructed with fragments having binding mode conservation (BM conservation) above 0.5 mTIS. However, stricter thresholds may lead to better/different results depending on the fragments of independent cases. Consequently, an additional threshold (BM conservation) has been introduced in the reconstruction pipeline as an option to restrict this feature even more when needed (Fig. 7C). Therefore, only fragments meeting the specified threshold will be used for the following compound’s reconstruction.
4. *Representative fragment’s fingerprint:* One unique fragment can be a substructure of multiple different compounds, which could be in multiple different PDB structures (or complexes). Therefore, for reconstruction purposes, it is necessary to define a consensus binding mode (interactions fingerprint) for a specific fragment. Such consensus has been constructed by aggregating all known fingerprints of a given fragment into one that contains the frequency of the observed non-covalent interactions. For instance, in (Fig. 7D) the example fragment is part of three different compounds. When constructing its representative binding mode, the fingerprints of each independent compound, are all merged into one by considering the frequency of the activated bins. In other words, the number of times is observed as active divided by the total number of fingerprints found for the fragment. It should be noted that the constructed representative fingerprint is not any more of the binary type but rather float due to the frequency score. Nonetheless, since it is based on real observations there is a direct correspondence between the consensus fingerprint and the interactions.
5. *Reconstructed compound’s fingerprint:* Finally, all the representative fingerprints describing the binding mode of each of the compound’s fragments are merged into one unique compound fingerprint (Fig. 7E). The merging of fingerprints is done by accounting for the union of all activated bins and calculating the mean between frequencies. The reconstructed fingerprint represents the binding mode of the compound to the target in question.

### Reconstruction quality evaluation

The quality of the reconstruction approach was evaluated in the PDB subset of 213106 compound-target pairs. The PDB subset includes all compound-target complexes found up to the second level (Ligand level) of the pyramid in Fig. 1, along with the non-fragmentable compounds that were excluded at the fragmentation stage. The reconstruction pipeline was applied to the PDB subset, trying different combination of the modifiable thresholds, i.e. the compound’s proportion and the fragments binding mode conservation (see Table 1). To avoid bias in the validation, the binding mode (fingerprint) of a compound-target complex is reconstructed without using the structural data from the complex itself. Meaning, the construction of representative binding mode of fragments does not take into consideration the fingerprint of the compound-target complex to be reconstructed. Furthermore, the quality of reconstruction was evaluated for each thresholds combination. To this purpose, a Reconstruction Similarity Score (RSS) was defined as the mean of all the reconstructed-original fingerprint pairs similarities, which was calculated using the simple Tanimoto Similarity approach.

Thus, the RSS was defined as follow:

2 $$\begin{aligned} RSS= \frac{\sum _{n=1}^{P}\left( \frac{O_{n}bins \cap R_{n}bins}{O_{n}bins+R_{n}bins-O_{n}bins\cap R_{n}bins}\right) }{P}, \end{aligned}$$

where, *P* is the total of reconstructed-original fingerprint pairs, $O_{n}bins$ is the activated bins in the original fingerprint, and $R_{n}bins$ the activated bins in the the reconstructed fingerprint.

### ChEMBL dataset

The ChEMBL dataset in SQLite format (v26 released in March 2020) was locally downloaded. Subsequently, the data retrieved was filtered according to the following criteria: compounds under the category of small molecules, compounds having SMILES descriptor and UniProt ids, compounds having activity data (type IC50, EC50, Kd, and Ki in nanomolar, compounds that are not in PDB, compounds binding and having activity data to PDB targets, and compounds with molecular weight < 600Da (see Additional file 6: Fig. S3 for more details).

### Generation of figures and plots

The RDKit (Version 2018.09.1) *Draw.MolToFile()* method was used to generate svg (scalable vector graphics) files of chemical structures. Plotting was done using the python package Matplotlib.Pyplot (Version 2.1.1) [29] with the *plot()*, *pie()*, *bar()* and *scatter()* methods. Figures 3 and 4 were generated with a 2D visualization tool provided by PharmaAI company. All figures were edited using Inkscape Vector Graphic Editor v1.0 (4035a4f, 2020-05-01).

## Conclusions

Given the conserved nature of the fragments binding mode, they have proved to be helpful to extend the limits of structure-based drug repositioning by offering a different perspective to explore the binding mode of drugs. The reconstruction turned out to be relatively successful as it fairly represents the relevant non-covalent interactions defining the binding mode of the reconstructed drugs. Although fragments tend to have a highly conserved binding mode, most of them never reach the perfect conservation, which comes hand to hand with the binding mode variability under particular binding environments. On the other hand, the compound proportion limitation reflects the still unmet necessity of a representative structural space covering a diverse set of molecular fragments. Nevertheless, in general, the approach was able to reconstruct multiple compound-target pairs at optimal thresholds and high similarity to the actual fingerprints, which calls for an optimistic future on the approach’s potential. The reconstructions are of great value and benefit to the structure-based drug repositioning since they automatically enlarge the technique’s scope and allow to explore the so far “unexplored compounds” from a structural perspective. Additionally, novel machine learning techniques could improve the conventional pattern matching screening by exploiting such reconstructed data. In a bigger picture, the building blocks concept allows the transfer of any other feature that may be relevant for the drug discovery process and that given to data limitations is not yet fully available.

## Supplementary information

**Additional file 1**. Fragment-target binding mode conservation: The file shows for each fragment-target pair thefragment’s InChiKey, the target’s UniProt ID, the minimum TIS, the maximum TIS, and the mean TIS observed in allavailable complexes

**Additional file 2**. PDB test data set with original and reconstructed fingerprints: The file shows for each compound-target complex in the PDB test dataset: the complex UID (PDB:HETID:CHAIN:POS), the compound’s SMILES, the target’s UniProt ID, the MW of the compound, the origianl interactions fingerprint, the reconstructed interactions fingerprint, and the fragments used for the reconstruction (according to the defined thresholds in the pipeline).

**Additional file 3**. ChEMBL data set with reconstructed fingerprints: The file shows for each compound-target complex in our ChEMBL dataset: the compound’s InChiKey, the target’s UniProt ID, and the reconstructed fingerprint.

**Additional file 4**. Cutoff selection of the fragments molecular weight: The figure shows in the Y-axis the number of PDB complexes (A) and the number of unique fragment such complexes (B) for each range of molecular weight in the X-axis.

**Additional file 5**. Number of different targets and compounds for the PDB fragments: The figure shows the scatter plot of all PDB fragments in terms of the number of targets they bind to (X-axis) and the number of superstructure compounds the are part of. The red box at the bottom left encloses the majority of fragments, having a mean of targets equal to 12.1 and a mean of different compounds of 7.8.

**Additional file 6**. ChEMBL compounds data set for reconstruction: All compounds in ChEMBL were extracted and filtered according to the reconstruction pipeline criteria, in order to build up a clean testing dataset to evaluate the performance of the novel introduced pipeline.

## Data Availability

All data sets, on which the conclusions of this manuscript rely, can be found as additional supporting files (more details on the Additional files section). The reconstruction pipeline can be found on https://github.com/madasme/FrInRecon.
