# Supplementary figures and images for: Decomposing compounds enables reconstruction of interaction fingerprints for structure-based drug screening
Source: J Cheminform. 2022 Mar 15;14:17. doi: 10.1186/s13321-022-00592-w (PMC8922937; doi:10.1186/s13321-022-00592-w)

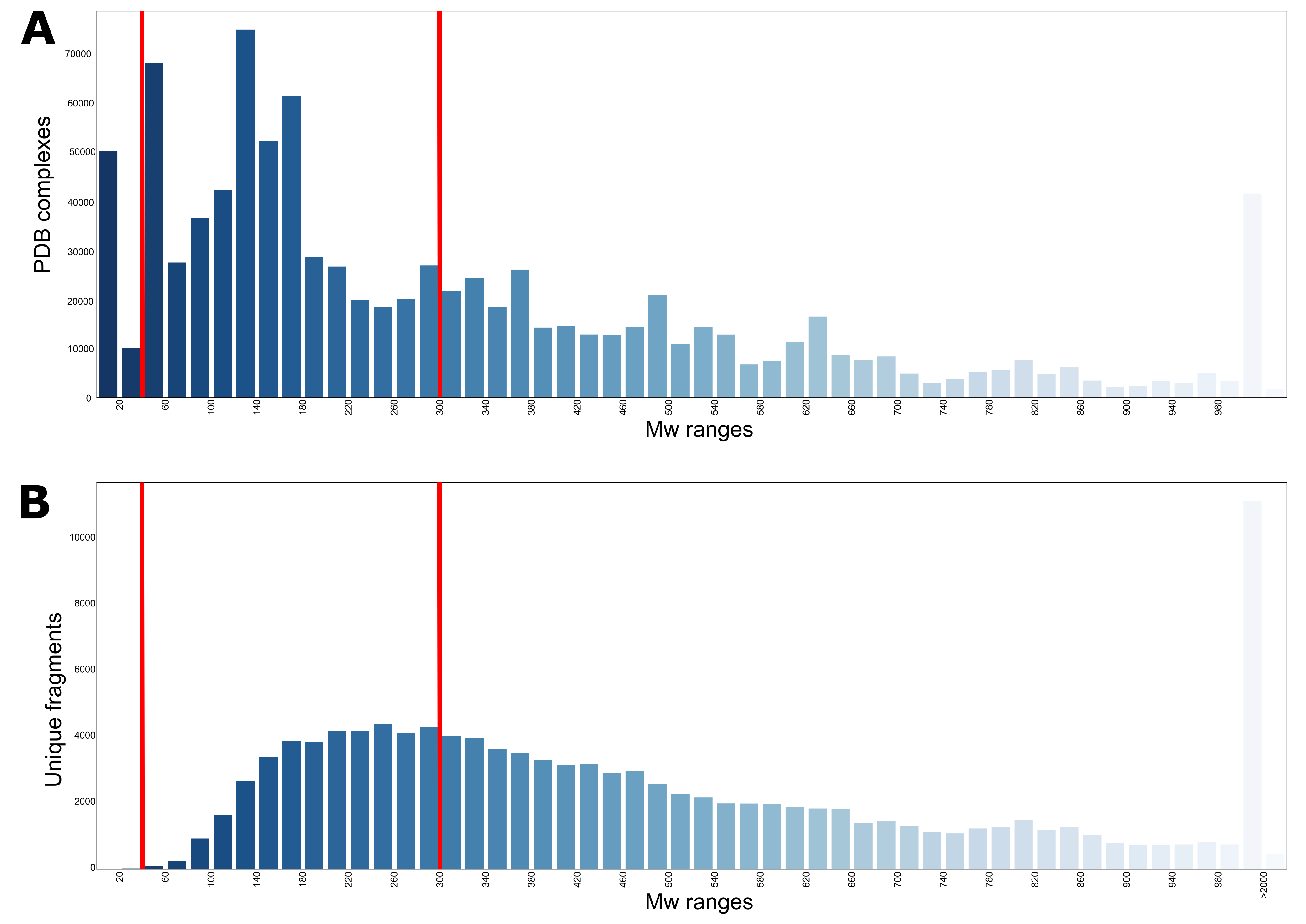

Supplement: Supplementary file 4 — Additional file 4. Cutoff selection of the fragments molecular weight: The figure shows in the Y-axis the number of PDB complexes (A) and the number of unique fragment such complexes (B) for each range of molecular weight in the X-axis. [file 13321_2022_592_MOESM4_ESM.png]

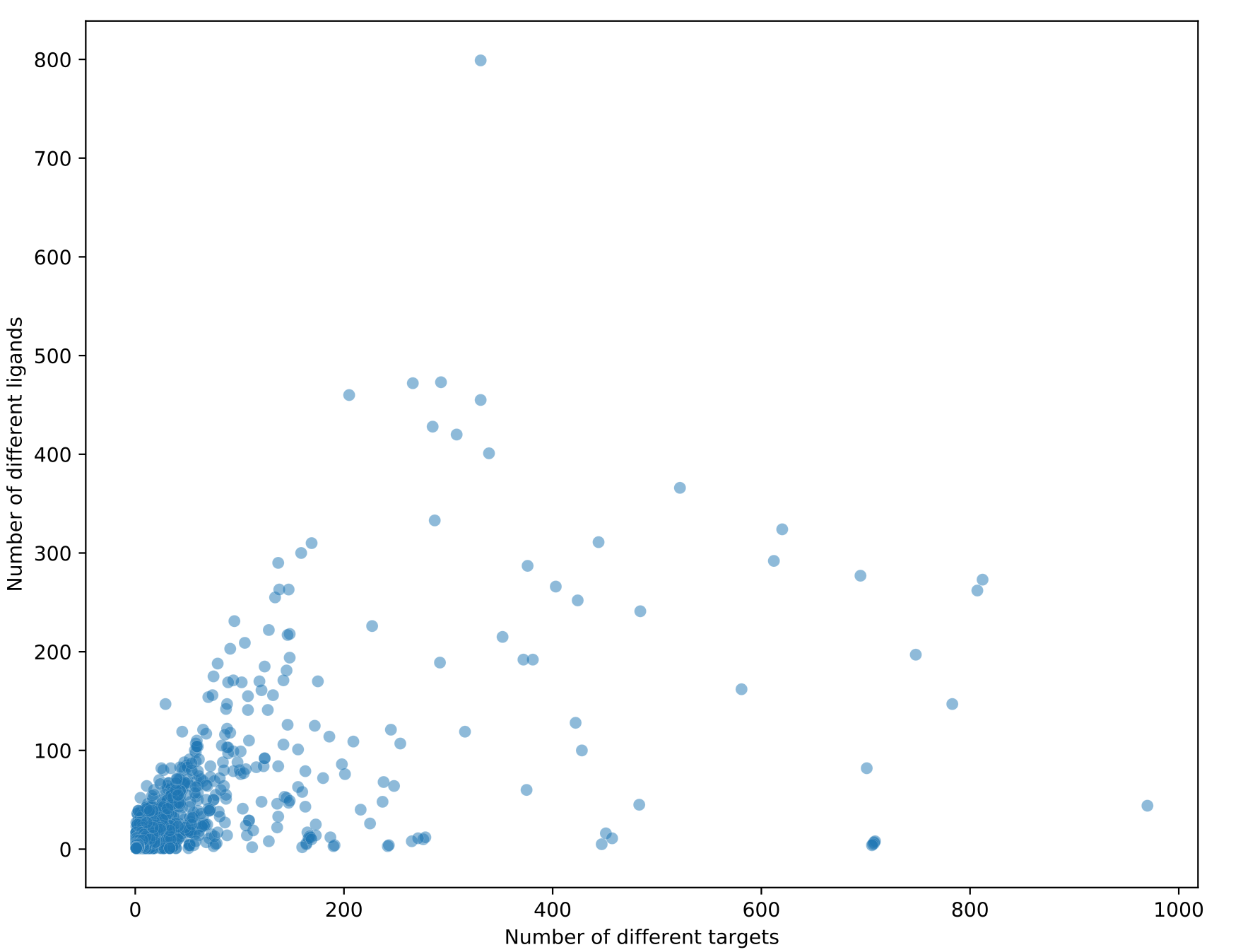

Supplement: Supplementary file 5 — Additional file 5. Number of different targets and compounds for the PDB fragments: The figure shows the scatter plot of all PDB fragments in terms of the number of targets they bind to (X-axis) and the number of superstructure compounds the are part of. The red box at the bottom left encloses the majority of fragments, having a mean of targets equal to 12.1 and a mean of different compounds of 7.8. [file 13321_2022_592_MOESM5_ESM.png]

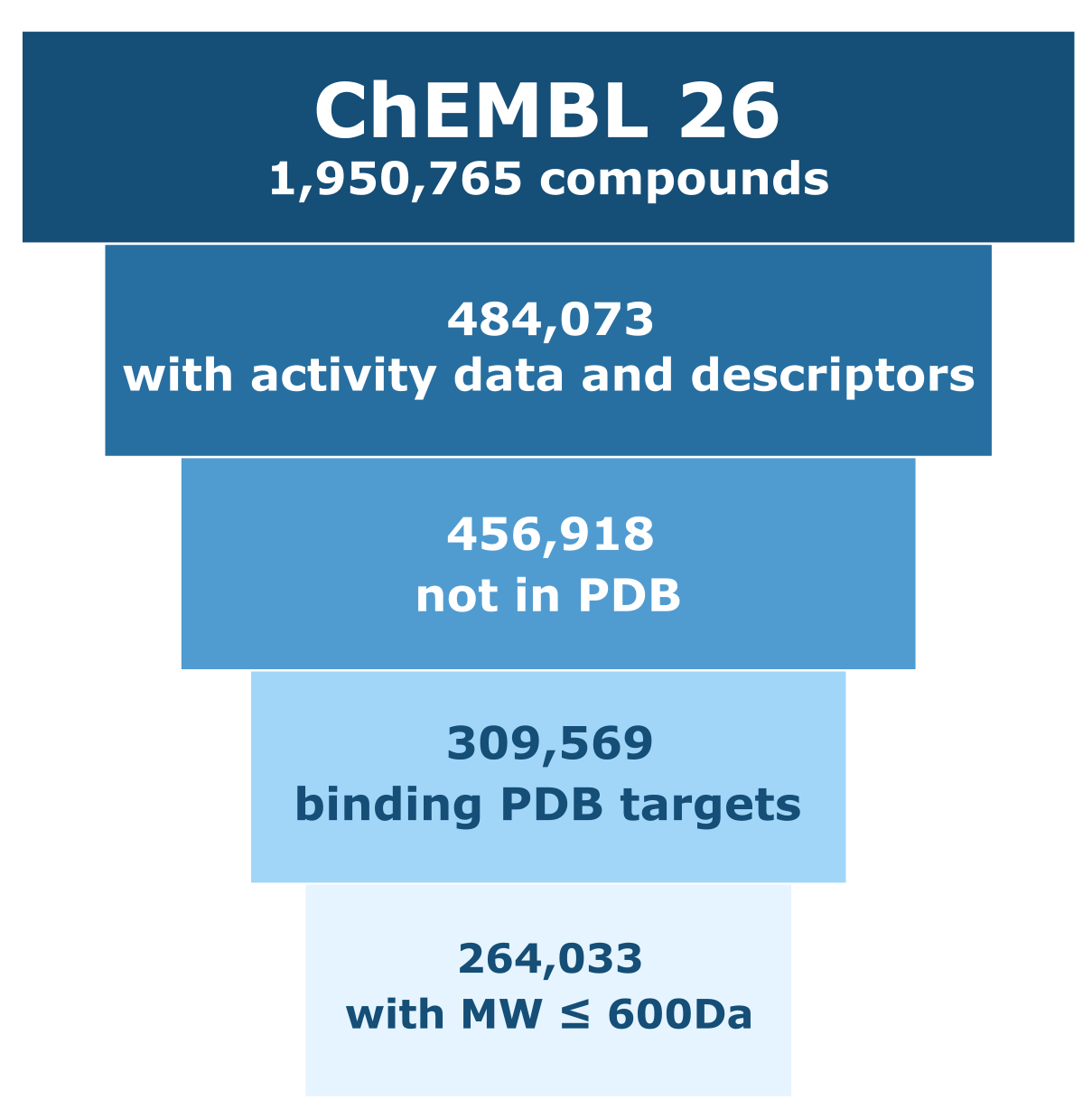

Supplement: Supplementary file 6 — Additional file 6. ChEMBL compounds data set for reconstruction: All compounds in ChEMBL were extracted and filtered according to the reconstruction pipeline criteria, in order to build up a clean testing dataset to evaluate the performance of the novel introduced pipeline. [file 13321_2022_592_MOESM6_ESM.png]
